# Fish Oil Alters the Metabolome, Antioxidative Potential, and Secretory Profile of Visceral Adipose Tissue in Mice with High-Fat Diet-Induced Obesity Compared with Other Dietary Fat Sources

**DOI:** 10.3390/molecules31050849

**Published:** 2026-03-04

**Authors:** Jacek Wilczak, Adam Prostek, Piotr Karpiński, Karolina Ciesielska, Żaneta Dzięgelewska-Sokołowska, Małgorzata Gajewska

**Affiliations:** 1 Institute of Veterinary Medicine, Department of Physiological Sciences, Warsaw University of Life Sciences, 02-787 Warsaw, Poland; adam_prostek@sggw.edu.pl (A.P.); karolina_ciesielska@sggw.edu.pl (K.C.); zaneta_dziegelewska-sokolowska@sggw.edu.pl (Ż.D.-S.); malgorzata_gajewska@sggw.edu.pl (M.G.); 2 Department of Technology and Food Safety, Faculty of Health Sciences, University of Lomza, 18-400 Lomza, Poland; pkarpinski@al.edu.pl

**Keywords:** adipose tissue, saturated fatty acids, polyunsaturated fatty acids, adiponectin, leptin, antioxidant capacity, superoxide dismutase, amino acids catabolism

## Abstract

Dietary fat quality, determined by fatty acid composition, plays a central role in regulating adipose tissue function and metabolic homeostasis in obesity. This study examined whether different dietary fat sources modulate the secretory activity, antioxidant capacity, and metabolomic profiles of visceral adipose tissue (VAT) in mice with established high-fat diet (HFD)-induced obesity. Male C57BL/6J mice were rendered obese by long-term feeding with a lard-based HFD and subsequently maintained on isocaloric HFDs containing lard, coconut oil, olive oil, or fish oil. Antioxidant capacity, redox enzyme activities, adipokine levels, and untargeted metabolomic profiles of VAT were analyzed. Fish oil-enriched HFD significantly improved antioxidant potential and partially restored redox enzyme activity compared with the lard-based diet. It preserved adiponectin levels and reduced leptin accumulation in VAT. Multivariate metabolomic analyses showed clear separation of dietary groups and distinct metabolic signatures related to fat quality. Replacement of lard with fish oil induced a coordinated remodeling of the lipid and amino acid metabolism and reduced metabolites linked to mitochondrial overload and oxidative stress, whereas saturated fat-rich diets promoted patterns consistent with metabolic dysfunction. These findings indicate that dietary fat quality reshapes adipose tissue metabolism in obesity and highlights fish oil as a strategy to attenuate adipose tissue dysfunction.

## 1. Introduction

Adipose tissue is now widely recognized as a central immunometabolic organ whose function extends far beyond passive energy storage. Under physiological conditions, adipose tissue contributes to metabolic homeostasis by regulating lipid fluxes, endocrine signaling, and immune tolerance through the balanced secretion of adipokines and lipid mediators [1]. In obesity, however, profound structural and functional remodeling of adipose tissue occurs, leading to the development of chronic low-grade inflammation that represents a hallmark of obesity and a major driver of its metabolic complications [2]. The initiation, persistence, and resolution of adipose tissue inflammation are critically shaped by both the quantity and the qualitative composition of fatty acids stored in and released from adipocytes, as well as by the capacity of the tissue to maintain redox homeostasis [3,4,5].

The onset of adipose tissue inflammation in obesity typically arises as a consequence of chronic energy excess and adipocyte hypertrophy. As adipocytes expand, local oxygen supply becomes insufficient, resulting in hypoxia, endoplasmic reticulum (ER) stress, mitochondrial dysfunction, and increased generation of reactive oxygen species (ROS). These stress signals induce the expression and secretion of pro-inflammatory adipokines and chemokines, including leptin, monocyte chemoattractant protein-1 (MCP-1), and interleukins, which promote immune cell recruitment [1,6]. Macrophage accumulation in adipose tissue increases significantly with obesity and is strongly correlated with systemic insulin resistance [3]. Recruited macrophages adopt a predominantly pro-inflammatory phenotype and amplify local cytokine production, establishing a self-reinforcing inflammatory microenvironment. In this early phase, adipose inflammation develops primarily as a downstream effect of lipid overload and cellular stress [7].

Fatty acid composition is a critical determinant of whether this inflammatory response resolves or progresses to a chronic pathological state. Saturated fatty acids (SFAs), particularly long-chain species (LC-SFAs), such as palmitic and stearic acid, are potent amplifiers of adipose tissue inflammation. Increased levels of circulating and locally released SFAs in obesity activate stress and innate immune signaling pathways in adipocytes and macrophages, including NF-κB- and JNK-mediated pathways, leading to increased expression of MCP-1 and pro-inflammatory interleukins. Experimental studies have shown that fatty acids can trigger inflammatory responses through Toll-like receptor 4 (TLR4)-dependent mechanisms, linking lipid excess directly to insulin resistance [8]. Although current evidence suggests that SFAs may act indirectly by altering membrane composition and lipid metabolism rather than serving as classical TLR4 ligands, the net effect remains a robust amplification of inflammatory signaling. In parallel, SFA-induced mitochondrial overload and impaired β-oxidation increase ROS production, further reinforcing redox-sensitive inflammatory pathways [9].

As inflammation becomes chronic, it transitions from being merely a consequence of obesity to a causal driver of further metabolic deterioration. Pro-inflammatory cytokines impair insulin signaling, increase adipocyte lipolysis, and elevate circulating non-esterified fatty acids, which exacerbate lipid overload in adipose tissue and peripheral organs. This establishes a vicious cycle in which inflammation sustains adipocyte dysfunction, promotes ectopic lipid deposition, and accelerates the progression of obesity-related metabolic diseases. In this context, adipose tissue inflammation actively contributes to the maintenance and worsening of the obese phenotype [10].

In contrast to SFAs, monounsaturated fatty acids (MUFAs)—most notably oleic acid—exert neutral or anti-inflammatory effects within adipose tissue. MUFAs improve lipid partitioning, reduce lipotoxic stress, and attenuate SFA-induced activation of inflammatory signaling pathways. Oleic acid has been shown to counteract palmitate-induced NF-κB and JNK activation, suppress MCP-1 expression, and preserve insulin sensitivity in adipocytes and macrophages [11]. Diets enriched in MUFAs are consistently associated with lower leptin levels, higher adiponectin concentrations, reduced macrophage infiltration, and improved redox balance, supporting adipose tissue metabolic flexibility [12,13]. MUFA-rich diets are associated with improved fat oxidation and overall metabolic flexibility, an effect partly mediated by lower leptin levels, indicating a more favorable adipose tissue metabolic profile [14].

Polyunsaturated fatty acids (PUFAs) have the most complex and context-dependent effects on adipose inflammation. N-6 PUFAs, particularly arachidonic acid, can give rise to pro-inflammatory eicosanoids when present in excess, whereas n-3 PUFAs —especially eicosapentaenoic acid (EPA) and docosahexaenoic acid (DHA)—are widely recognized for their anti-inflammatory and pro-resolving properties. EPA and DHA suppress the expression of MCP-1 and interleukins, promote macrophage polarization towards anti-inflammatory phenotypes, and increase adiponectin levels. Crucially, they serve as substrates for the biosynthesis of specialized pro-resolving mediators, including resolvins, protectins, and maresins, which actively terminate inflammation and restore tissue homeostasis [15]. However, obesity is frequently associated with impaired incorporation and metabolism of PUFAs within adipose tissue, limiting the generation of these resolution signals and contributing to a failure to resolve inflammation [16].

In obesity, adipose tissue inflammation arises initially as an adaptive response to excess lipid storage, but evolves into a chronic pathological process that both reflects and drives metabolic dysfunction. Fatty acid quality and redox homeostasis constitute an integrated regulatory system governing the trajectory of adipose tissue inflammation. Preserving antioxidant capacity is a prerequisite for harnessing the full immunometabolic benefits of PUFAs and for preventing the transition of adipose inflammation from a transient adaptive response into a self-perpetuating driver of obesity-related metabolic disease.

The present study aimed to investigate whether qualitative differences in dietary fat sources, under conditions of long-term high-fat feeding, modulate adipokine secretion, redox homeostasis, and the metabolomic profile of visceral adipose tissue in established obesity. We used an experimental model of diet-induced obesity (DIO) in which C57BL/6J mice were exposed for 27 weeks to a high-fat diet (HFD) containing 45% kcal from fat. In the first stage of the experiment (weeks 1–15) obesity was induced by feeding mice with a lard-based HFD. Subsequently, in the second stage of the dietary intervention, which was the main part of the experiment lasting 12 consecutive weeks, mice received HFD, but differing in the main source of lipids: lard (HFD-L), coconut oil (HFD-CO), olive oil (HFD-OO) or fish oil (HFD-FO). This study investigated how diets based on commonly used dietary fats: lard, coconut oil, olive oil, or fish oil (rich in EPA and DHA) differentially affect antioxidant defense mechanisms, secretory activity, and the key metabolic pathways in the visceral adipose tissue (VAT). This integrated approach was designed to elucidate system-level mechanisms linking dietary fat quality with adipose tissue dysfunction in obesity.

## 2. Results

### 2.1. Analysis of Oxidative Stress Markers in Murine Visceral Adipose Tissue

**Figure 1:**
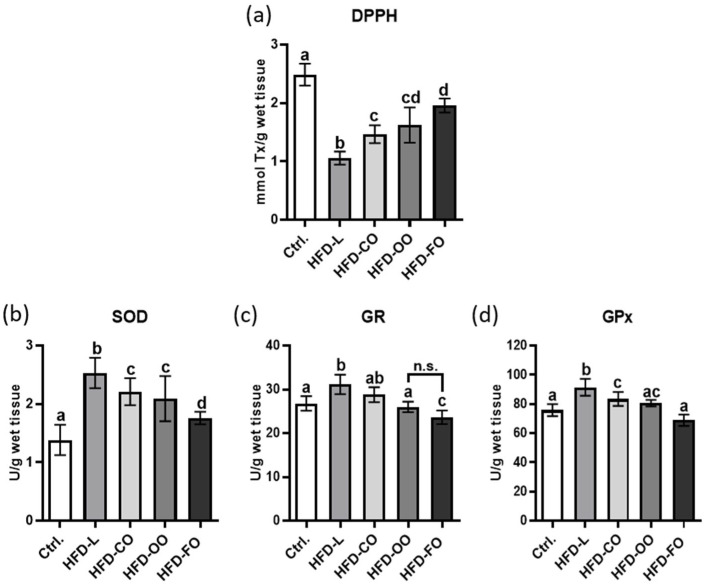
Effect of different high-fat diets on markers of antioxidant capacity of murine visceral adipose tissue: concentration of reduced DPPH (2,2′-diphenyl-1-picrylhydrazyl radical) (**a**), superoxide dismutase (SOD) activity (**b**), glutathione reductase (GR) activity (**c**), glutathione peroxidase (GPx) activity (**d**). Experimental groups included: control, high-fat diet with lard (HFD-L), coconut oil (HFD-CO), olive oil (HFD-OO), and fish oil (HFD-FO). Bars represent mean values ± SD. Different letters above the bars indicate statistically significant differences between groups (*p* < 0.05), as determined by one-way ANOVA followed by Tukey’s post hoc test. Abrreviation n.s. symbolizes not significant difference.

To identify differences among the experimental groups—the control group (Ctrl.) and the high-fat diet groups (HFD-L, HFD-FO, HFD-CO, and HFD-OO)—Tukey’s multiple comparisons test based on least squares (LS) means was applied (Figure 1).

In the DPPH assay that was used to analyze the antioxidant capacity of VAT, all comparisons between the control group and the experimental groups showed statistically significant differences (Figure 1a). Visceral adipose tissue samples isolated from control animals, receiving standard feed for rodents (with 10% of energy from fat) throughout the entire experiment, showed the highest antioxidant capacity among all analyzed groups. On the contrary, VAT samples from the HFD-L group, in which mice were fed a high-fat diet containing lard as the main source of dietary fat, showed the lowest value of this parameter among all analyzed experimental groups (*p* < 0.0001). Other HFD groups also showed significantly lower values than the control group (*p* < 0.0001); however, VAT samples from the HFD-FO group showed the highest antioxidant capacity among all HFD groups. The only comparison that did not reach statistical significance was that between the HFD-FO and HFD-OO groups (*p* = 0.3148).

The study also analyzed the activity of chosen antioxidant enzymes (Figure 1b–d). Superoxide dismutase (SOD) activity showed the highest value in the VAT samples from HFD-L group and the lowest in the control group (*p* < 0.0001) (Figure 1b). SOD activity was also significantly increased in the HFD-CO and HFD-OO groups compared to control (*p* < 0.0001), but lower than in the HFD-L group (*p* = 0.0361; *p* = 0.0032, respectively). SOD activity in HFD-FO was the lowest among all HFD groups (*p* < 0.05), although significantly higher than in the control (*p* = 0.0475). For glutathione reductase (GR), statistically significant differences were observed between the control group and the HFD-L group that showed the highest activity of this enzyme (*p* = 0.0015) (Figure 1c). In contrast, the GR activity in the HFD-FO group was significantly lower than in the control (*p* = 0.0275), and reduced compared to the HFD-L and HFD-CO groups (*p* < 0.0001; *p* = 0.0002, respectively). GR activity did not differ significantly between the HFD-FO and HFD-OO groups. Glutathione peroxidase (GPx) activity in VAT from control mice was significantly lower than in the HFD-L and HFD-CO groups (*p* < 0.0001; *p* = 0.00395, respectively) (Figure 1d). In contrast, GPx activity in the VAT from HFD-FO and HFD-OO groups did not differ significantly from the control group. Among VAT samples from all HFD groups, the adipose tissue samples in the HFD-L group showed significantly higher GPx activity compared to the other high-fat diets (*p* < 0.05).

### 2.2. Concentration of Chosen Adipokines in Murine Visceral Adipose Tissue

The data representing adipokine levels in the murine visceral adipose tissue were analyzed using the same statistical approach as for oxidative stress markers.

**Figure 2:**
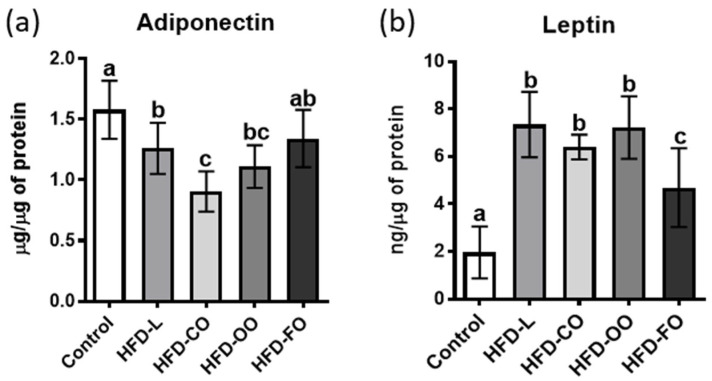
Effect of different high-fat diets on adiponectin (**a**) and leptin (**b**) levels in murine VAT, expressed as μg/μg of total protein (adiponectin) and ng/μg of total protein (leptin). Experimental groups included: control, high-fat diet with lard (HFD-L), coconut oil (HFD-CO), olive oil (HFD-OO), and fish oil (HFD-FO). Bars represent mean values ± SD. Different letters above the bars indicate statistically significant differences between groups (*p* < 0.05), as determined by one-way ANOVA followed by Tukey’s post hoc test.

Adiponectin concentration in the VAT of control mice was significantly higher than in the HFD-L, HFD-CO and HFD-CO groups (*p* < 0.01), but did not differ significantly from the HFD-FO group (*p* = 0.0818) (Figure 2a). Leptin concentration was the lowest in the VAT of mice in the control group and differed significantly from all HFD groups *p* < 0.0001) (Figure 2b). In contrast, no significant differences were detected among the HFD-L, HFD-CO and HFD-OO groups. Interestingly, the level of leptin in VAT samples from the HFD-FO group was significantly lower compared to other HFD groups (*p* < 0.01), although still significantly higher than in the control group (*p* < 0.0001).

**Figure 3:**
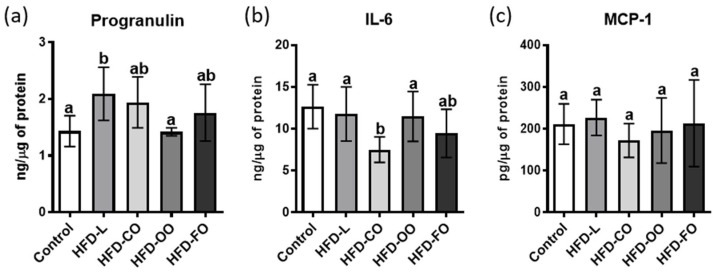
Effects of different high-fat diets on progranulin (**a**), IL-6 (**b**) and MCP-1 (**c**) levels in murine VAT, expressed as ng/μg of total protein (progranulin and interleukin 6) and pg/μg of total protein (MCP-1). Experimental groups included: control, high-fat diet with lard (HFD-L), coconut oil (HFD-CO), olive oil (HFD-OO), and fish oil (HFD-FO). Bars represent mean values ± SD. Different letters above the bars indicate statistically significant differences between groups (*p* < 0.05), as determined by one-way ANOVA followed by Tukey’s post hoc test.

In addition, chosen adipokines responsible for the immune response regulation were analyzed (Figure 3). The concentration of progranulin was the lowest in the control group, but did not differ significantly from the HFD-CO, HFD-OO and HFD-FO groups (Figure 3a). The highest level of progranulin was detected in the HFD-L, and differed significantly from the control group (*p* = 0.0156) and the HFD-OO group (*p* = 0.0134). Comparable levels of interleukin 6 (IL-6) were observed in the visceral adipose tissue of the animals from the control, HFD-L, and HFD-OO groups (Figure 3b). A significantly lower concentration was recorded only in the HFD-CO group when compared to the control group (*p* = 0.0006), and to the HFD-L and HFD-OO groups (*p* = 0.0056; *p* = 0.0174, respectively). The HFD-FO group showed an intermediate IL-6 concentration that was not significantly different from the control, HFD-L, HFD-OO or the HFD-CO group. MCP-1 concentrations in adipose tissue did not differ significantly among the experimental groups (Figure 3c). Comparable levels were observed in the control group and in all HFD groups.

### 2.3. Metabolic Profiles of the Visceral Adipose Tissue in Mice Fed High-Fat Diets Containing Different Lipid Sources

Untargeted metabolomic analyses were conducted to assess changes in metabolomic profiles induced by qualitative differences in dietary fat composition, using pairwise comparisons between the control group and individual high-fat diet (HFD) groups, as well as between selected HFD variants. In particular, detailed analyses focused on comparing the metabolomic profiles of mice fed a lard-based high-fat diet (HFD-L) with those receiving a fish oil-enriched high-fat diet (HFD-FO), enabling assessment of metabolic effects specifically related to fat source.

**Figure 4:**
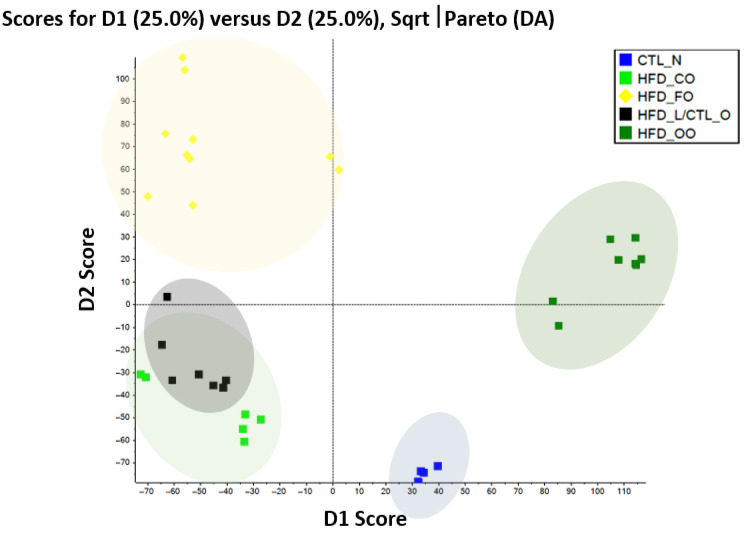
Principal components analysis (PCA) of visceral adipose tissue metabolome in mice from the control group (Ctrl_N) and experimental groups fed a high-fat diet differing in the lipid source: lard (HFD_L), coconut oil (HFD_CO), olive oil (HFD_OO), and fish oil (HFD_FO).

Metabolomic data were mean-centered and appropriately scaled prior to multivariate statistical analysis. Principal Component Analysis (PCA) was applied to explore global differences in metabolomic profiles among experimental groups. A *p* value ≤ 0.05 was considered statistically significant. PCA of the visceral adipose tissue metabolome revealed a clear separation of experimental groups based on the dietary fat source, indicating substantial remodeling of the metabolic profile in response to different lipid compositions (Figure 4). The control group clustered distinctly from all high-fat diet (HFD) groups along the first principal component (PC1), which accounted for the largest proportion of total variance, reflecting the strong impact of high-fat feeding on adipose tissue metabolism. Among HFD-fed animals, further segregation was observed depending on the type of dietary fat. Mice fed the lard-based high-fat diet (HFD-L) formed a separate cluster, partially overlapping with the coconut oil-based diet (HFD-CO), but clearly shifted from the control group, suggesting a metabolomic signature characterized by pronounced lipid overload and metabolic stress. In contrast, diets enriched with fish oil (HFD-FO) and olive oil (HFD-OO) resulted in distinct clustering away from the HFD-L, indicating that unsaturated fatty acid sources substantially modulated adipose tissue metabolic pathways. Notably, the HFD-FO group showed the greatest divergence from HFD-L along PC2, consistent with a unique metabolomic profile associated with n-3 PUFA-derived metabolites and altered lipid mediator pathways. The coconut oil-based diet (HFD-CO) occupied an intermediate position between HFD-L and unsaturated fat-enriched groups, suggesting partial metabolic differentiation likely related to the distinct chain length and metabolic handling of medium-chain saturated fatty acids.

Metabolites that differed significantly between the HFD-L and HFD-FO groups were subjected to pathway analysis using MetaboAnalyst 6.0, integrating pathway enrichment with pathway topology analysis. Identified metabolites were subsequently mapped to KEGG pathways to facilitate biological interpretation. Comparative analysis revealed pronounced differences in metabolic pathway representation between the HFD-L and HFD-FO groups, indicating that dietary fat source markedly influences systemic metabolism (Table 1). The most affected pathways included selenoamino acid metabolism, represented by metabolites such as serine and gamma-glutamyl-Se-methylselenocysteine, as well as intermediates of small-molecule alcohol metabolism, including 2,3-butanediol. Significant differences were also observed in carbohydrate-related pathways, including fructose and mannose metabolism, galactose metabolism, hexose phosphorylation, and starch and sucrose metabolism. These pathways involved multiple hexoses and sugar alcohols, such as D-glucose (α- and β-anomers), D-fructose, D-mannose, L-fucose, sorbitol, and galactitol, indicating substantial remodeling of carbohydrate handling depending on the dietary fat source. Pathways related to glycan turnover, including N-glycan degradation, keratan sulfate degradation, and sialic acid metabolism, were also identified in the HFD-L vs. HFD-FO comparison. These pathways were represented by metabolites such as D-galactose, L-fucose, and myo-inositol, suggesting differences in glycoprotein and glycolipid metabolism between dietary interventions. Alterations in amino acid-related metabolism were evident as well, particularly in glycine, serine, alanine, and threonine metabolism, as well as in the urea cycle and amino group metabolism. Metabolites such as creatine, creatinine, dimethylglycine, γ-aminobutyric acid (GABA), and threonine contributed to the differentiation of these pathways. Additional differences were detected in tryptophan metabolism, butanoate metabolism, and the pentose phosphate pathway, represented by metabolites including deoxyribose and 6-phosphoglucono-D-lactone. At the pathway level, the comparison between the HFD-L and HFD-FO groups was characterized by coordinated alterations in carbohydrate metabolism, amino acid turnover, redox-related pathways, and glycan processing, indicating broad metabolomic remodeling induced by substitution of lard with fish oil in a high-fat diet.

A complementary analysis compared the HFD-FO group with all other HFD groups combined (HFD-L, HFD-CO, and HFD-OO) (Table 2). This approach enabled identification of metabolic features specifically associated with fish oil supplementation while minimizing effects common when feeding with a high-fat diet. This comparison revealed a distinct metabolomic signature dominated by lipid metabolism–related pathways, particularly de novo fatty acid biosynthesis and fatty acid activation. These pathways were characterized by multiple saturated and unsaturated fatty acids—including palmitic, myristic, oleic, α-linolenic, and γ-linolenic acids—as well as medium-chain acyl-CoA derivatives and adenosine monophosphate, reflecting differences in fatty acid synthesis and activation. Further enrichment analysis highlighted alterations in saturated fatty acid β-oxidation and linoleate metabolism, involving metabolites such as decanoyl-CoA, palmitic acid, lipid peroxidation-related aldehydes, and glutathione-associated intermediates. In addition, inflammation-related lipid mediator pathways, including leukotriene metabolism and prostaglandin biosynthesis from arachidonate, were uniquely represented in this comparison, suggesting differential regulation of eicosanoid-related processes in response to fish oil-derived lipids. Central carbon metabolism pathways—including glycolysis, gluconeogenesis, pyruvate metabolism, and the pentose phosphate pathway—also contributed to group discrimination, alongside differences in butanoate metabolism and amino acid-related pathways. Furthermore, pathways associated with membrane and signaling lipid metabolism, such as phosphatidylinositol phosphate metabolism and glycosphingolipid metabolism, were identified.

Collectively, these results demonstrated that fish oil supplementation within a high-fat diet is associated with a distinct systemic metabolomic signature dominated by lipid metabolism-related pathways, accompanied by coordinated changes in energy metabolism, redox regulation, and amino acid turnover.

**Figure 5:**
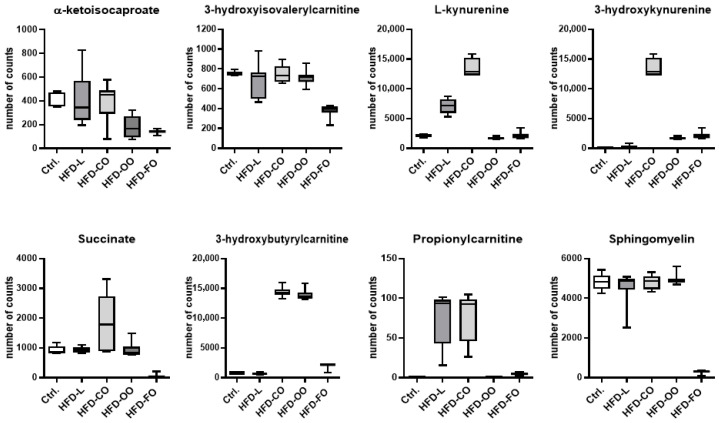
Number of metabolite features identified as discriminating the VAT metabolome of mice from the control group and from high-fat diet-fed groups containing different lipid sources: lard (HFD-L), coconut oil (HFD-CO), olive oil (HFD-OO), and fish oil (HFD-FO). Bars represent mean values ± SD.

Selection of metabolites for subsequent targeted metabolomic analysis was guided by the results of the preceding untargeted analysis, which aimed to capture global metabolic changes induced by dietary interventions, as evidenced by PCA. The untargeted approach enabled identification of metabolites and metabolic pathways that most strongly differentiated experimental groups, providing a rational basis for targeted compound selection. Metabolites selected for targeted analysis met the following criteria: statistical significance in group comparisons; involvement in key metabolic pathways showing coordinated network-level alterations; unambiguous chemical identification with known molecular mass reflected in ionization data; and potential functional relevance to lipid, carbohydrate, and amino acid metabolism, redox regulation, and inflammatory responses. Accordingly, subsequent analyses focused on eight metabolites: α-ketoisocaproate, 3-hydroxyisovalerylcarnitine, 3-hydroxyisobutyrylcarnitine, succinate, L-kynurenine, 3-hydroxykynurenine, propionylcarnitine, and sphingomyelin (Figure 5). Metabolomic analysis revealed differences in the counts of individual metabolites in mouse visceral adipose tissue across all examined groups, indicating significant diversification of metabolic profiles in response to the applied dietary interventions. The biological roles of these compounds are discussed in detail in Section 3.

## 3. Discussion

Excessive dietary fat intake is one of the major factors that hinder weight reduction, impedes recovery from obesity, and promotes the development of its associated comorbidities [17,18]. However, an increasing body of scientific evidence indicates that, in this context, not only the quantity but also the quality of dietary fat plays a critical role [19,20]. Therefore, in the present study we investigated whether diverse fat sources in high-fat diets could alter the secretory function and metabolic activity of visceral adipose tissue. For the experiment, C57BL/6J mice were used—a strain that is highly susceptible to obesity when subjected to a high-fat diet. The in vivo study was divided into two phases. The aim of the first phase was to induce obesity in the animals serving as the experimental model. This was achieved by feeding the mice for 15 weeks with a commonly described high-fat diet (HFD) providing 45% of total energy from fat, with lard as the primary lipid source. At the end of this phase, mice in the HFD group exhibited markedly increased body weight, elevated blood glucose concentrations, and significantly higher levels of stearic and oleic acids in visceral adipose tissue, accompanied by a reduction in linoleic acid content in this tissue compared with control animals. These data have been published in our earlier article, describing in details the diet-induced obesity model used in our study [21]. In the second part of the experiment, mice from the HFD group were subdivided into four smaller groups that continued to receive a high-fat diet for an additional 12 weeks. The total fat content was identical across all groups, but the primary lipid sources differed. The experimental diets were enriched with one of the following fat sources: lard, coconut oil, olive oil, or fish oil (cod liver oil). Analysis of the fatty acid profile in the visceral adipose tissue showed a close correspondence with the fatty acid composition of the respective experimental diets [21]. Mice in all HFD groups showed a significantly increased adipocyte size in the visceral adipose tissue, proving the development of VAT hypertrophy. Body mass in animals in all high-fat diet groups remained elevated compared with the control group throughout the entire second phase of the study; however, mice fed the high-fat diet supplemented with fish oil exhibited a slightly slower rate of body-weight gain than those receiving the other high-fat diets [21]. These data, described in detail in our previous article [21], correspond well with the values of the feed conversion ratio (FCR) presented in Table A1. FCR values were the highest in control mice and significantly lower in animals fed the high-fat diets: HFD-L, HFD-CO and HFD-OO. Lower FCR observed in mice in the HFD groups reflects increased efficiency of body weight gain under high-fat feeding conditions. Animals fed the fish-oil-based HFD (HFD-FO) also showed lower FCR value compared to the control group, but the difference was not significant (Table A1). When the FCR values where compared among the HFD group, no significant differences were noted. Our experiment showed that mice fed the fish oil-based HFD in the second part of the experiment exhibited lower efficiency of body weight gain compared with the other HFD groups, under conditions of long-term high-fat feeding. Interestingly, by the end of the dietary intervention blood glucose concentration remained elevated only in the HFD-L group but normalized in other HFD groups [21].

The composition of dietary fat is a key determinant of adipose tissue function, systemic metabolic regulation, and the risk of obesity-related disorders. Visceral adipose tissue is an active metabolic depot that contributes to systemic inflammation and insulin resistance through adipokine and cytokine secretion [22,23]. In this study, we examined how high-fat diets differing in fat source influence the levels of major adipokines in the visceral adipose tissue of obese mice. Adiponectin and leptin are central regulators of energy balance, insulin sensitivity, and inflammatory responses [24,25]. In obesity, reduced adiponectin and elevated leptin are strongly associated with insulin resistance, chronic low-grade inflammation, and metabolic complications [23]. Our results showed that the lard-based HFD (rich in long-chain SFAs) led to a significant reduction in adiponectin levels in VAT compared to the control group, which was consistent with numerous animal and human studies [24,26,27]. This effect could be driven by direct inhibition of adiponectin gene transcription by palmitate, increased oxidative and ER stress, and enhanced degradation of adiponectin, leading to impaired secretion [28,29]. It was also shown that high SFAs intake activates the TLR4/NF-κB signaling pathway, increasing pro-inflammatory cytokine expression and promoting chronic VAT inflammation, which further suppresses adiponectin synthesis [30]. The lowest concentration of this adipokine among all HFD groups was observed in the HFD-CO group. Similar findings have been reported in other studies. Both short- and long-term administration of a high-fat diet supplemented with coconut oil has been shown to reduce circulating levels of this protein and the expression of its gene in adipose tissue. Studies using the 3T3-L1 cell model demonstrated that fatty acids characteristic of coconut oil (e.g., lauric acid) likewise decrease adiponectin expression [31,32]. In contrast, the experimental group fed a diet supplemented with fish oil (rich in long-chain n-3 polyunsaturated fatty acids, primarily EPA and DHA) was the only group in which adiponectin levels in the adipose tissue of obese mice were comparable to those observed in the lean control mice. This finding is consistent with previous reports showing that diets enriched in n-3 PUFAs increase circulating adiponectin and induce *Apn* transcription in the visceral adipose tissue, and these effects are at least partly mediated by PPARγ activation [33]. Recent studies further indicate that fish oil supplementation mitigates HFD-induced adipose tissue dysfunction by modulating gene expression, the oxylipin profile, and epigenetic marks associated with inflammation and adipocyte function [34,35].

The study also assessed the effect of experimental diets on leptin concentration in visceral adipose tissue. It was shown that obesity and HFD consistently increase leptin production, which is accompanied by the development of leptin resistance, weakening its physiological effects and contributing to further weight gain and metabolic disturbances [36,37,38]. The results of our study are consistent with these observations. All high-fat diets contributed to an increase in the concentration of this protein in VAT. However, it should be emphasized that the fish-oil-based HFD caused the lowest increase in the leptin level detected in the visceral adipose tissue among all tested high-fat diets. Similar results were also obtained by other research groups. In animal models, adding fish oil to a high-fat diet (HFD) was shown to reduce leptin expression in visceral depots [34]. Moreover, clinical studies in humans revealed that EPA/DHA supplementation lowered plasma leptin concentrations, particularly in populations with visceral obesity or in older adults. Rausch et al. reported that administration of 2.5 g/day of EPA + DHA for eight weeks decreased leptin while simultaneously increasing adiponectin, improving the leptin/adiponectin ratio [39]. Comparable findings were obtained by Hernandez et al., where fish oil supplementation reduced inflammation in adipose tissue and normalized leptin signaling [40].

This study also evaluated the effect of high-fat diets containing different fat sources on the concentration of MCP-1, interleukin 6 (IL-6), and progranulin in the visceral adipose tissue. The chosen proteins belong to the group of adipokines that regulate the immune response. Surprisingly, the analyses did not show any marked differences in the levels of these adipokines between the control group and the HFD groups. Such findings may indicate that the dietary intervention, despite its duration, did not induce a fully established chronic inflammatory state within VAT. Alternatively, the absence of consistent changes could reflect methodological limitations, as the analytical sensitivity might not have detected subtle but biologically relevant alterations. These results highlight the complexity of the adipose tissue response to nutritional interventions and suggest that MCP-1, IL-6, and progranulin may not serve as sensitive markers of dietary fat quality under conditions of long-term high-fat feeding.

The present study also evaluated parameters of the antioxidant capacity of the visceral adipose tissue. Obesity induced by the lard-based HFD led to pronounced disturbances in redox homeostasis in VAT, as evidenced by both the results of the DPPH assay and changes in the activity of the antioxidant enzymes. The reduced antioxidant potential measured by DPPH in the HFD-L group compared with the control group indicates a diminished overall free-radical scavenging capacity, a characteristic feature of obesity associated with excessive intake of SFAs. This phenomenon is well documented in the literature: excessive SFAs supply promotes mitochondrial dysfunction in adipocytes, increases electron leakage from the respiratory chain, and enhances the generation of ROS, ultimately resulting in a secondary overload of enzymatic antioxidant defense systems [41,42].

Alterations in the activities of glutathione reductase (GR) and glutathione peroxidase (GPx) observed in the visceral adipose tissue of mice in the HFD-L group indicate disruption of the glutathione axis, a key buffering system against oxidative stress. Reduced or inadequately regulated activity of these enzymes in obesity has repeatedly been described as a consequence of chronic oxidative stress accompanied by persistent low-grade inflammation [43]. Importantly, the absence of marked changes in the concentration of the inflammatory markers (MCP-1, IL-6) in VAT samples from the HFD-L group suggests that redox imbalance precedes the development of a fully established inflammatory response. This observation is consistent with the concept of “metabolic oxidative stress” as an early pathogenic driver of obesity-related dysfunction [44].

Replacing lard with fish oil (HFD-FO) significantly altered the antioxidant response profile in VAT. Compared with the HFD-L group, a diet enriched with long-chain n-3 PUFAs, i.e. EPA and DHA, resulted in an improvement in the antioxidant capacity (DPPH) and significant changes in the antioxidant enzyme activities, particularly superoxide dismutase (SOD) and GR. This effect can be interpreted in the context of the well-established biological properties of long-chain n-3 PUFAs. Despite their high susceptibility to lipid peroxidation, EPA and DHA modulate the expression of genes involved in antioxidant defense, among others, through activation of Nrf2-dependent pathways, improvement of mitochondrial function, and attenuation of pro-oxidant NF-κB signaling pathway [45,46,47]. The differences in SOD activity observed in the present study between the HFD-FO and HFD-L groups are consistent with reports showing that diets rich in EPA/DHA may restore the balance between superoxide anion production and its dismutation, limiting secondary oxidative damage to lipids and proteins in adipocytes [41]. In parallel, the lack of significant differences in GPx activity between the control and HFD-FO group suggests fish oil stabilizes the functioning of the glutathione axis, preventing its secondary overload, which is typically observed in obesity induced by SFA-rich diets. This phenomenon has been previously described as part of an adaptive redox response under conditions of n-3 PUFA supply [48].

The absence of significant differences in GR activity between the control and HFD-CO group suggests that coconut oil does not induce overload of the glutathione axis as severely as lard; however, it does not provide full redox protection. A significant difference in GPx activity between the control and HFD-CO group indicates selective activation of peroxide-neutralizing mechanisms, which may result from the specific properties of medium-chain SFAs. These fatty acids are oxidized more rapidly and stored to a lesser extent in adipocytes. Coconut oil induces partial redox adaptation, mitigating certain aspects of the oxidative stress compared with lard, but does not exhibit a profile as favorable as that observed with EPA/DHA-enriched diets.

In contrast, a diet containing olive oil displayed an antioxidant profile intermediate between HFD-FO and HFD-CO. Antioxidant capacity (DPPH) differed significantly from HFD-L while not differing significantly from HFD-CO, suggesting a moderate improvement in antioxidant capacity compared with lard. Regarding antioxidant enzymes, the absence of significant differences in GR activity between the control and HFD-OO group indicates relative preservation of glutathione homeostasis. In parallel, significant differences between HFD-L and HFD-OO in the activity of GR and GPx in VAT suggest that monounsaturated fatty acids present in olive oil attenuated SFA-induced redox overload, although they did not fully normalize the oxidative stress parameters. Functionally, olive oil promotes the stabilization of antioxidant responses by limiting excessive activation of ROS-neutralizing enzymes, yet its effect remains weaker than that of diets enriched in long-chain n-3 PUFAs.

When dietary interventions incorporating different fatty acid sources are compared in mice with previously induced obesity, the results indicate that fatty acid composition, rather than fat quantity alone, determines the nature of the oxidative response in VAT. A lard-based HFD favors accumulation of oxidative stress and dysregulation of the antioxidant enzymes, whereas diets containing the long-chain n-3 PUFAs promote a more balanced redox adaptation, even under conditions of high energy intake.

In this study, metabolomic profiling enabled the identification of metabolites involved in pathways regulating oxidative stress and inflammation in visceral adipose tissue. The discussion below summarizes how selected metabolites may contribute to metabolic disturbances in obesity.

α-Ketoisocaproate (KIC), a product of leucine transamination, reflects disrupted branched-chain amino acid (BCAA) metabolism characteristic of obesity and insulin resistance. Elevated α-keto acids indicate impaired BCAA oxidation and enhanced lipogenesis, processes linked to mTOR activation and pro-inflammatory signaling in adipocytes and macrophages, contributing to chronic metaflammation [49]. Increased levels of carboxylated BCAA derivatives, such as 3-hydroxyisovalerylcarnitine and 3-hydroxybutyrylcarnitine, indicate mitochondrial dysfunction and disturbances in acylcarnitine metabolism. Their accumulation is associated with impaired β-oxidation, excess energy substrates, and elevated ROS production, all of which promote oxidative stress and inflammation in adipose tissue [49,50]. Altered propionylcarnitine concentrations reflect disruptions in short-chain acyl metabolism and the metabolic integration of BCAA and SFA pathways. Increased levels suggest a shift toward acylcarnitine accumulation, characteristic of mitochondrial overload and oxidative stress in obesity and insulin resistance [51].

Succinate, a TCA cycle intermediate, acts not only as a metabolic substrate but also as a signaling molecule. By activating SUCNR1 (GPR91), succinate enhances lipolytic and immune signaling. Under metabolic stress, increased succinate promotes pro-inflammatory macrophage activation and cytokine release, contributing to obesity-associated inflammation [52].

Kynurenine pathway metabolites (L-kynurenine, 3-hydroxykynurenine) arise from tryptophan catabolism driven by cytokine-regulated enzymes such as IDO1. Their elevation in obesity reflects chronic inflammation and immune activation [53,54]. Some downstream metabolites directly generate ROS and modulate transcription factors involved in oxidative and inflammatory responses [30].

Sphingomyelins—key membrane lipids—represent strong metabolomic markers of obesity. Their increased levels, along with ceramide derivatives, promote oxidative stress, ROS generation, and pro-inflammatory signaling, contributing to insulin resistance and metabolic dysfunction [55].

Metabolomic alterations observed in the present study indicate that BCAA-derived metabolites signal mitochondrial overload and metabolic stress, succinate amplifies inflammatory pathways, kynurenine metabolites integrate immune and oxidative responses, and sphingomyelins link lipid signaling with redox imbalance. Together, they form a metabolic signature of oxidative stress and chronic inflammation in obese adipose tissue.

Metabolite differences were quantified based on count intensities. Among all HFD groups, these metabolites—except for L-kynurenine and 3-hydroxykynurenine, whose levels were similar to those in control, HFD-L, and HFD-OO groups—were present at the lowest counts in the visceral adipose tissue of mice fed fish oil (HFD-FO), corresponding to increased dietary intake of EPA and DHA. Dietary sources of EPA and DHA have metabolite-specific effects on adipose tissue biochemical networks. Preclinical, and some clinical, studies showed that n-3 PUFAs reduce tissue ceramide burden and beneficially remodel sphingolipid pools, contributing to decreased lipotoxic signaling and amelioration of oxidative stress [56,57]. Effects of EPA/DHA on BCAA-derived metabolites (α-ketoisocaproate, 3-hydroxyisovalerylcarnitine, 3-hydroxybutyrylcarnitine, propionylcarnitine) are heterogeneous across studies: fish oil induces shifts in acylcarnitine profiles consistent with altered peroxisomal and mitochondrial fatty acid oxidation but does not uniformly reverse markers of incomplete BCAA oxidation in obesity [57,58]. N-3 PUFA can modulate TCA cycle intermediates, including succinate, with differential effects of EPA versus DHA on TCA flux reported in metabolomic studies, underscoring context-dependent effects on succinate signaling [59,60]. Finally, modulation of the kynurenine pathway by n-3 PUFA has been observed in some interventional studies—particularly in inflamed or stressed cohorts—but not others, indicating that suppression of IDO1-driven kynurenine/tryptophan elevation by EPA/DHA may occur only when a pro-inflammatory drive is present at baseline [61,62]. Collectively, these data argue that EPA and DHA reshape adipose tissue metabolomes in a metabolite- and context-specific manner rather than inducing uniform normalization of all obesity-associated metabolomic perturbations.

To further contextualize the functional relationships among the identified metabolites, the network-based interpretation proposed above can be directly anchored to the multivariate structure revealed by PCA and other metabolomic analyses. The clear separation of experimental groups observed in the PCA space indicates that the combined variation of BCAA-derived metabolites, acylcarnitines, TCA intermediates, tryptophan catabolites, and sphingolipids represents a coordinated metabolic response rather than isolated biochemical changes [63]. Importantly, the clustering pattern suggests these metabolites act synergistically to define distinct adipose tissue metabolic phenotypes associated with dietary fat composition and obesity-related stress [64].

The strong contribution of α-ketoisocaproate together with 3-hydroxyisovalerylcarnitine and 3-hydroxybutyrylcarnitine to group discrimination in PCA is consistent with their central role in shaping the metabolic variance captured by the first principal component. In the heatmap, these metabolites cluster tightly, showing shared regulation and a common biochemical origin in impaired BCAA catabolism [65]. Their coordinated elevation in specific dietary groups supports the interpretation that disrupted mitochondrial handling of BCAA constitutes a dominant axis of metabolic remodeling in obese adipose tissue [66]. This pattern aligns with the notion that BCCA overload and incomplete oxidation form an early metabolic signature that precedes overt inflammatory activation [64].

The positioning of propionylcarnitine alongside hydroxy-acylcarnitines in both the heatmap clustering and PCA loadings further reinforces the concept of incomplete substrate oxidation. Their joint contribution to group separation suggests that alterations in acylcarnitine profiles are not random but reflect a systemic shift toward mitochondrial overload [63]. In PCA space, these metabolites contribute to variance along both PC1 and PC2, indicating that they integrate signals from amino acid catabolism and lipid oxidation pathways. This dual contribution underscores their role as metabolic intermediates linking BCAA metabolism with fatty acid β-oxidation and redox imbalance.

Succinate emerges as a metabolite with high discriminatory power in the PCA and a distinct positioning in the heatmap relative to upstream TCA intermediates. This pattern suggests accumulation rather than simple flux enhancement through the TCA cycle. Its contribution to group separation supports the interpretation that succinate functions as a metabolic bottleneck and signaling molecule under conditions of mitochondrial stress [67]. The PCA results capture succinate not merely as an energetic intermediate, but as a node integrating metabolic overload with low-grade immunometabolic signaling, consistent with its known role in redox-sensitive and inflammatory pathways.

The clustering of L-kynurenine and 3-hydroxykynurenine, together with their moderate but consistent contribution to PCA separation, shows coordinated activation of the kynurenine pathway across dietary groups. Their positioning suggests that alterations in tryptophan metabolism represent a secondary axis of metabolic variance, likely downstream of mitochondrial stress and oxidative imbalance [68]. The presence of these metabolites in the PCA loadings supports the concept that redox-dependent immune modulation contributes to the adipose tissue phenotype, even in the absence of strong cytokine-driven inflammation.

Sphingomyelins display a distinct clustering pattern and contribute to PCA separation in a manner that reflects their role as structural and signaling lipids rather than transient metabolic intermediates [69]. Their separation from acylcarnitines and BCAA-derived metabolites indicates a complementary but stabilizing function within the metabolic network. In PCA space, sphingomyelins help define group identity along axes associated with membrane remodeling, insulin sensitivity, and lipid-mediated stress signaling. This suggests that sphingolipid remodeling acts to merge and maintain the altered metabolic state revealed by the more dynamic amino acid and acylcarnitine profiles.

Taken together, the PCA and the differences between the concentrations of individual metabolites in the visceral adipose tissue provide strong empirical support for the proposed network model. Metabolites associated with BCAA catabolism and acylcarnitine accumulation dominate the primary axis of variation, indicating mitochondrial overload as the central driver of adipose tissue metabolic remodeling. Succinate and kynurenine pathway metabolites occupy intermediate positions, linking energy metabolism to redox and immune signaling. Sphingomyelins form a lipid-based regulatory layer that stabilizes the dysfunctional phenotype. This hierarchical organization explains why metabolic and redox alterations are more prominently captured by multivariate analyses than classical inflammatory markers.

Importantly, the PCA-driven separation of the dietary groups demonstrates that qualitative differences in fat sources reshape this metabolomic network in a coordinated manner. Rather than inducing uniform inflammation, different lipid profiles modulate the balance between mitochondrial stress, redox adaptation, and lipid signaling, resulting in distinct but related metabolic phenotypes. Thus, the multivariate metabolomic patterns observed in this study not only corroborate individual metabolite changes but also reveal the system-level architecture underlying adipose tissue dysfunction in obesity.

## 4. Materials and Methods

### 4.1. Animals and Diet Intervention

The present study was based on an in vivo experiment performed on 72 male C57BL/6J mice (6 weeks old) purchased from Charles River Laboratories (Sulzfeld, Germany). The experiment was conducted at the animal house of the Department of Immunology Medical University of Warsaw. All procedures were approved by the 2nd Local Ethics Committee in Warsaw (Resolution No. WAW2/081/2022; date of approval: 6 July 2022) in accordance with the Polish law and the EU Directive 2010/63/EU for animal experiments. Detailed description of the animal study experimental design was published in our previous article [21]. Briefly, animals were housed in groups of 6 mice per cage in a temperature-controlled room under a 12 h light–dark cycle with ad libitum access to water and food. After one week of acclimatization to the animal house conditions mice were randomly separated into two main groups: experimental group (*n* = 54)—mice fed a high-fat diet (HFD) containing 45% of energy (kcal) from fat (40% kcal from lard, 5% kcal from soybean oil), control group (*n* = 18)—mice fed a standard purified feed for mice, comprising 10% of energy from fat (6% kcal from soybean oil, 4% kcal from lard). The first part of the dietary intervention, aiming to induce overweight/obesity in mice in experimental group was carried out for 15 weeks. The remaining animals from the experimental group were divided into 4 HFD groups (12 animals each) and fed high-fat diet comprising different sources of fat, as follows: HFD-L group—mice fed a high-fat diet containing 40% kcal from lard, 5% kcal from soybean oil; HFD-CO—mice fed a high-fat diet containing 30% kcal from coconut oil, 10% kcal from lard, 5% kcal from soybean oil, HFD-OO—mice fed a high-fat diet containing 30% kcal from olive oil, 10% kcal from lard, 5% kcal from soybean oil, HFD-FO—mice fed a high-fat diet containing 30% kcal from fish oil (cod liver oil), 10% kcal from lard, 5% kcal from soybean oil. Control group (Ctrl., *n* = 12) continued to receive the control standard purified feed. All diets described in this section were produced by ZooLab (Sędziszów, Poland), following the nutritional recommendation for rodents. The composition of all diets is shown in detail in Table 3. The second part of dietary intervention was carried out for 12 consecutive weeks. Feed conversion ratio (FCR) was calculated for each mouse as the ratio of cumulative feed intake to body weight gain over the 12-week (84 days) intervention (the second part of experiment). Daily feed intake was assumed to be 2.8 g/mouse/day for the second part experiment based on the average consumption recorded for the cohort; therefore, cumulative intake was estimated rather than individually measured. The cumulative feed intake per mouse (2.8 g/mouse/day) was multiplied by the duration of the experiment’s second part, giving 235.2 g feed per mouse. Body weight gain was calculated as the difference between body weight measured at the beginning and end of the second part (ΔBW = BW in week 27 − BW in week 15). Thus, FCR = (2.8 × 84)/(BW in week 27 − BW in week 15) and is expressed as g feed per g body weight gain (dimensionless ratio). Results of the FCR are presented in Table A1 in the Appendix A. Mice showing weight loss (ΔBW ≤ 0) by the end of experiment were excluded from FCR analysis. One animal from the control group, one from the HFD-CO and HFD-OO groups and two animals from the HFD-FO group were excluded. After the final week of the experiment the mice were anesthetized through intraperitoneal injection with a solution of ketamine (87 mg/kg of bod mass) and xylazine (7.5 mg/kg of body mass), and an intracardiac puncture was immediately performed to collect fresh blood samples. The mice were sacrificed by cervical dislocation. The abdominal visceral white adipose tissue (VAT) samples were collected by snap freezing in liquid nitrogen until further analyses.

### 4.2. Fatty Acids Composition in the Diet

The fatty acid composition was determined using gas chromatography (GC), according to the standards outlined in the following norms: PN-ISO 5509:2001 (Fats and oils—Preparation of methyl esters of fatty acids), PN-EN-ISO 5508:1996 (Analysis of methyl esters of fatty acids by gas chromatography), and the method described by Hara and Radin [70].

Lipid extraction from feed samples was performed using a hexane–isopropanol solvent system (3:2, *v*/*v*) according to the method of Hara and Radin [61]. Subsequent preparation of fatty acid methyl esters (FAMEs) was carried out in accordance with PN-ISO 5509:2001.

The analysis of FAMEs was conducted using a GCMS-QP2020 NX system (Shimadzu, Kyoto, Japan) equipped with an AOC-6000 RTC autosampler and an SH-2330 capillary column (30 m × 0.25 mm i.d., 0.25 μm film thickness). The injector temperature was set at 250 °C. The oven temperature was programmed to start at 40 °C for 1 min, increase at 15 °C/min to 150 °C, and then rise at 8 °C/min to 240 °C, with a final hold of 3 min. Helium was employed as the carrier gas at a constant flow rate of 1.60 mL/min and a split ratio of 1:10. The transfer line and ion source were both maintained at a temperature of 250 °C. The mass spectrometer was set to total ion current (TIC) mode and programmed to scan masses from 50 to 500 Da.

The identification of selected fatty acids was tentative and based on retention times and mass spectral similarity. Fatty acids were identified by comparing their mass spectra and retention times with reference standards (NCP-GLC-674, NCP-GLC 37, NCP-GLC 96, NCP-GLC490, NU-Chek-Prep Inc., Elysian, MN, USA). The results were expressed as the percentage of individual fatty acids in the total lipid content (Table 4).

### 4.3. Oxidative Stress Markers in the Murine Visceral Adipose Tissue

Visceral adipose tissue was homogenized in phosphate-buffered saline (PBS), vortexed for 15 min, and centrifuged at 500× *g* for 15 min. The supernatants were used for analyses. Glutathione reductase (GR) and glutathione peroxidase (GPx) were measured in VAT samples using the commercial Randox reagent kit (catalog number GR2368 and RS505, respectively). DPPH (2,2′-diphenyl-1-picrylhydrazyl radical) scavenging method was used as an additional analytical method, in addition to the measurement of the total antioxidant status (TAS) and thiobarbituric acid reactive substances (TBARS), the results of which were presented in our earlier publication [21]. DPPH assay was performed according to the method developed by Brand-Williams et al. (1995) [4] with some modifications by assumptions and recommendations by Kedare and Singh (2011) [5]. Briefly, the assay was performed on VAT extracted with methanol (100 µL of VAT homogenate with 250 µL of methanol). After extraction and vortexing the methanol layer was collected and used in further analysis. A solution of 1 mM DPPH in 80% (*v*/*v*) methanol was stirred for 40 min. Absorbance of the solution was adjusted to 0.650 at 517 nm using fresh 80% (*v*/*v*) methanol. Then, 50 µL of standard or sample were mixed with 2.95 mL of DPPH solution and incubated for 30 min in the dark. Decrease of absorbance was monitored at 517 nm after 30 min. Control samples consisted either of 50 µL of distilled deionized water in 2.95 mL of DPPH solution (for Trolox standard), or 50 µL of 50% (*v*/*v*) methanol in 2.95 mL of DPPH solution for samples. Analysis of the levels of oxidative stress markers were performed using at least 6 samples from each experimental group.

### 4.4. Analysis of Adipokine Concentration in the Murine Visceral Adipose Tissue

To extract protein, 300 mg of visceral adipose tissue was homogenized in Pierce™ RIPA buffer (cat. no. 89900, Thermo Fisher Scientific, Waltham, MA, USA) containing protease (P8340) and phosphatase (P5726) inhibitor cocktails (Merck/Sigma-Aldrich, Darmstadt, Germany). Next, the homogenates were kept on ice for 30 min and then centrifuged at 20,000× *g* for 30 min. The supernatants obtained after centrifugation, containing the extracted proteins, were carefully collected. Total protein concentration was determined using the Bio-Rad Protein Assay Dye Reagent according to the manufacturer’s instructions (Bio-Rad Laboratories Inc., Hercules, CA, USA). The level of adipokines in samples was quantified using a competitive specific enzyme immunoassay (ELISA) following the manufacturer’s protocol (Adiponectin—Mouse Adiponectin ELISA, cat. no. RD293023100R, BioVendor, Brno, Czech Republic; Leptin—Mouse and Rat Leptin ELISA, cat. no. RD291001200R, BioVendor, Brno, Czech Republic; MCP-1—Mouse MCP-1 ELISA, cat. no. RAF080R, BioVendor, Brno, Czech Republic; Interleukin 6—Mouse Interleukin 6 ELISA, cat. no. RAF071R, BioVendor, Brno, Czech Republic; Progranulin—PicoKine TM Quick ELISA Kit, cat. no. FEK1192, Boster Biological Technology, Pleasanton, CA, USA). Adipokine levels were assessed in at least 6 samples from each experimental group.

### 4.5. Metabolomic Analysis

Metabolomic analysis was performed using a high-pressure liquid chromatography Symbiosis Pico UHPLC system. The detector used for this analysis was a SCIEX TripleTOF 5600+ DuoSpray Source for SCIEX TripleTOF 5600+ (TurboIonSpray and APCI). At least 10 samples from each experimental group were analyzed. The acquired data were analyzed using SCIEX MarkerView™, XCMSplus, and MetaboAnalyst 6.0 software for comprehensive interpretation and extraction of metabolomic information.

The samples of VAT were homogenized in PBS and mixed with a mixture of acetonitrile and methanol in a 1:1 ratio. Samples were vortexed (2000 rpm for 15 min) and centrifuged for 15 min at 20,000× *g*. Supernatants were transferred to glass autosampler vials, and placed in an autosampler at 4 °C. Samples were injected directly into a Spark Holland Symbiosis™ Pico system. Chromatographic separation was performed on the Hypersil chromatographic column, BDS C18, (150 × 4.6 mm, 5 μm) with a Hypersil C18 guard column (10 × 2.1 mm, size 5 μm). The mobile phase consisted of methanol:formic acid (99:1, *v*/*v*) A and water:formic acid (99:1, *v*/*v*) B, the flow rate was constant at 500 µLmin^−1^. The gradient elution of the mobile phase 100% A was started at 1.1–40 min linear gradient to 100% B, 40.1–55 min 100% B, and 55.1–60 min linear gradient to 100% A.

LS/MS parameters: the optimized detection conditions included curtain gas (N_2_) 25 psi, nebulizer gas (N_2_) 20 psi, heater gas (N_2_) 50 psi, ion source voltage floating 5500 V, and source temperature 500 °C. Samples with a heated electrospray 3 ionization probe were measured in positive ionization (H-ESI+).

### 4.6. Statistical Analysis

Statistical analysis was performed using GraphPad PrismTM version 9.00 software (GraphPad Software, Inc., La Jolla, CA, USA). Normality of data was analyzed with the Shapiro–Wilk test. One-way analysis of variance (ANOVA) with Tukey’s multiple comparison post-test was used to determine the significance of effects between the experimental groups. Values are expressed as mean ± standard deviation (SD). Letters (a, b, c…) indicate significant differences between the specified groups, with *p* < 0.05.

The metabolite profiles, obtained within the 100–1100 Da range with a sensitivity of 5 cps, were analyzed using SCIEX MarkerView™ software. Comparative assessments between groups were conducted using Student’s *t*-tests and principal component analysis (PCA). The generated metabolomic profiling data sets were processed by the control software of the SCIEX Analyst^®^ mass spectrometer and saved in a specific data format (*.raw). The first step was to convert data from Excalibur-specific raw files to open format files (*.mzXML) using MS Convertor software (MSConvert version 1.5.2). Subsequently, the metabolomic data were processed using the XCMSplus platform. Additionally, PCA resulted in comparative profiles of metabolomes of specific groups, that were further analyzed by SCIEX MarkerView™ software. The classification of identified metabolites within particular metabolic pathways was conducted using the XCMSplus platform and MetaboAnalyst 6.0 software with a probability threshold of *p* < 0.0001. The identification of metabolites was carried out based on the ChemSpider database (accessed via SCIEX PeakView™). Indications above an 80% probability of confirming a given structure were compared with the indications of metabolites from the MetaboAnalyst 6.0.

## 5. Conclusions

This study demonstrates that long-term lard-based HFD-induced obesity leads to marked functional and metabolic remodeling of visceral adipose tissue, affecting adipokine secretion, redox homeostasis, and metabolomic architecture. Notably, oxidative imbalance—characterized by reduced antioxidant capacity and dysregulated antioxidant enzymes—appears in adipose tissue even without a fully developed pro-inflammatory cytokine response, supporting the concept that oxidative and metabolic stress are early pathogenic events preceding overt inflammation.

The quality of dietary fat critically influenced how adipose tissue responded. Diets enriched in long-chain n-3 PUFAs (EPA and DHA) promoted a more adaptive phenotype, preserving adiponectin levels, attenuating leptin overexpression, stabilizing the glutathione-dependent antioxidant system, and generating a metabolomic profile consistent with reduced mitochondrial overload. In contrast, lard- and coconut oil-based HFDs were associated with impaired adiponectin secretion, greater redox disruption, and metabolomic signatures indicative of incomplete β-oxidation and metabolic stress. Olive oil exerted intermediate effects, partially mitigating oxidative stress and adipokine dysregulation without fully restoring adipose tissue function.

Metabolomic analyses revealed coordinated remodeling of interconnected pathways involving BCAA catabolism, acylcarnitine accumulation, TCA cycle intermediates, kynurenine pathway activation, and sphingolipid metabolism. Multivariate analyses (PCA and heatmap clustering) confirmed that these metabolites form an integrated network reflecting mitochondrial overload, redox imbalance, and immunometabolic signaling. BCAA-derived metabolites and acylcarnitines dominated the primary axis of variance; succinate and kynurenine metabolites linked energy metabolism with redox and immune signaling, while sphingomyelins contributed a stabilizing lipid-signaling layer associated with insulin resistance and chronic dysfunction.

Overall, qualitative differences in dietary fat sources reshaped adipose tissue biology across multiple regulatory levels—from adipokine secretion and antioxidant defense to system-level metabolic organization. Enrichment of HFD with EPA and DHA emerged as an effective strategy to modulate these interconnected pathways, limiting oxidative and metabolic stress and promoting a more adaptive adipose phenotype despite sustained high-fat feeding.

## Figures and Tables

**Table 1:** List of metabolites differentiating metabolomes of mice VAT from the HFD-FO vs. HFD-L groups, assigned to individual metabolic pathways (Metaboanalyst 6.0).

Pathway	Symbol	Name
Selenoamino acid metabolism	C00065	Serine
	C05695	Gamma-Glutamyl-Se-methylselenocysteine
	C00265	2,3-Butanediol
Fructose and mannose metabolism	C01019	L-Fucose
	C00095	D-Fructose
	C00159	D-Mannose
	C00031	D-Glucose
	CE3074	Fructose-3-phosphate
	C00794	Sorbitol
	C00267	alpha-D-Glucose
Keratan sulfate degradation	C00124	D-Galactose
	C01019	L-Fucose
3-Chloroacrylic acid degradation	C06613	trans-3-Chloroallyl aldehyde
	C16348	cis-3-Chloroallyl aldehyde
N-Glycan degradation	C01019	L-Fucose
	C00159	D-Mannose
	C00124	D-Galactose
Galactose metabolism	C00124	D-Galactose
	tag1p-D	D-Tagatose-1-phosphate
	C00159	D-Mannose
	C01697	Galactitol
	C00031	D-Glucose
	C00795	D-Tagatose
	C00794	Sorbitol
	C02336	beta-D-Fructose
	C00221	beta-D-Glucose
	C00267	alpha-D-Glucose
	C00095	D-Fructose
Hexose phosphorylation	C00159	D-Mannose
	C00031	D-Glucose
	C00095	D-Fructose
	C00794	Sorbitol
	C00738	D-Gulose
Starch and sucrose metabolism	CE2839	Maltodecaose
	C00031	D-Glucose
	C00221	beta-D-Glucose
	C00267	alpha-D-Glucose
Glycine serine alanine and threonine metabolism	C01026	Dimethylglycine
	CE2026	3-methylcrotonoylglycine
	C00300	Creatine
	C00065	Serine
	C05519	L-Allothreonine
	C00188	L-Threonine
	C02218	2-Aminoacrylic acid
Pyrimidine metabolism	C01801	Deoxyribose
	C05145	3-Aminoisobutanoic acid
	C00881	Deoxycytidine
Phosphatidylinositol phosphate metabolism	C00124	D-Galactose
	C01204	myo-Inositol hexakisphosphate
	C00137	myo-Inositol
Butanoate metabolism	C06006	(S)-2-Aceto-2-hydroxybutanoic acid
	C00334	gamma-Aminobutyric acid
Pentose phosphate pathway	C01801	Deoxyribose
	C01236	6-Phosphonoglucono-D-lactone
	C00221	beta-D-Glucose
Tryptophan metabolism	C05645	4-(2-Amino-3-hydroxyphenyl)-2,4-dioxobutanoic acid
	C00272	Tetrahydrobiopterin
	C00936	alpha-D-Mannose
Sialic acid metabolism	C01582	Galactose
	C00221	beta-D-Glucose
	C00065	Serine
	C00267	alpha-D-Glucose
	C00124	D-Galactose
	C00137	myo-Inositol
Urea cycle/amino group metabolism	C00791	Creatinine
	C01756	Thiopurine
	C00300	Creatine
	C00334	gamma-Aminobutyric acid

**Table 2:** List of metabolites differentiating metabolomes of mice VAT from the HDF-FO group vs. other HFD groups (HFD-L, HFD-CO and HFD-OO combined), assigned to individual metabolic pathways (Metaboanalyst 6.0).

Pathway	Symbol	Name
De novo fatty acid biosynthesis	C00020	Adenosine monophosphate
	C00712	Oleic acid
	C06427	alpha-Linolenic acid
	C06426	gamma-Linolenic acid
	C06424	Myristic acid
	C00249	Palmitic acid
	C05274	Decanoyl-CoA (n-C10:0CoA)
Fatty acid activation	C00020	Adenosine monophosphate
	C01712	Elaidic acid
	C06427	alpha-Linolenic acid
	C06426	gamma-Linolenic acid
	C06424	Myristic acid
	C06423	Caprylic acid
	C00249	Palmitic acid
Phosphatidylinositol phosphate metabolism	C00124	D-Galactose
	C00033	Acetic acid
	C00137	myo-Inositol
	C00249	Palmitic acid
Leukotriene metabolism	C00020	Adenosine monophosphate
	CE2056	20-dihydroxyleukotriene B4
	C05356	5(S)-Hydroperoxyeicosatetraenoic acid
	CE5343	6,7-dihydro-5-oxo-12-epi-leukotriene B
	CE5944	10,11-dihydro-12-oxo-LTB4
	CE5179	6,7-dihydro-5-oxo-leukotriene B4
	C02165	Leukotriene B4
	C05952	Leukotriene E4
	CE2446	6E-12-epi-leukotriene B4
	C00051	Glutathione
	CE2445	6-trans-leukotriene B4
Butanoate metabolism	C00020	Adenosine monophosphate
	C00431	5-Aminopentanoic acid
	C06006	(S)-2-Aceto-2-hydroxybutanoic acid
	C00033	Acetic acid
Glycolysis and gluconeogenesis	C00020	Adenosine monophosphate
	C00033	Acetic acid
	C00031	D-Glucose
	C00221	beta-D-Glucose
	C00267	alpha-D-Glucose
Pyruvate metabolism	C03451	S-Lactoylglutathione
	C00051	Glutathione
	C00033	Acetic acid
Linoleate metabolism	CE2061	Acetylcarnosine
	CE6415	11-HpODE
	CE6506	3,4-epoxynonanal
	CE5922	3(S),10(R)-OH-octadeca-6-trans-4,12-cis-trienoate
	C00051	Glutathione
	CE2006	4-hydroxy-2-nonenal
	C06426	gamma-Linolenic acid
Glycosphingolipid metabolism	C00124	D-Galactose
	C00249	Palmitic acid
	C00270	N-Acetylneuraminate
	C06124	Sphingosine 1-phosphate
	C00962	beta-D-Galactose
	C00031	D-Glucose
	C01582	Galactose
Pentose phosphate pathway	C01801	Deoxyribose
	C00020	Adenosine monophosphate
	C06222	Sedoheptulose 1-phosphate
	C00221	beta-D-Glucose
	C05382	D-Sedoheptulose 7-phosphate
Glycine, serine, alanine and threonine metabolism	C00020	Adenosine monophosphate
	C03451	S-Lactoylglutathione
	C00719	Betaine
	C00300	Creatine
	C00051	Glutathione
	C00033	Acetic acid
Keratan sulfate degradation	C00124	D-Galactose
	C00270	N-Acetylneuraminate
Alkaloid biosynthesis II	C00020	Adenosine monophosphate
	C00180	Benzoic acid
Saturated fatty acids beta-oxidation	C05274	Decanoyl-CoA (n-C10:0CoA)
	C00249	Palmitic acid
Drug metabolism— other enzymes	C16622	N,N′-Diacetylhydrazine
	C02380	Mercaptopurine
Histidine metabolism	C00020	Adenosine monophosphate
	C00051	Glutathione
	C00135	Histidine
	CE2065	Acetylcarnosine
Valine, leucine and isoleucine degradation	C00020	Adenosine monophosphate
	C00183	L-Valine
	C03465	3-Methyl-2-oxopentanoate
	C00233	Ketoleucine
Prostaglandin formation from arachidonate	C00959	Prostaglandin B1
	C04686	Prostaglandin C1
	C00051	Glutathione
	C00020	Adenosine monophosphate
	CE1447	11-dehydrothromboxane B2
	C04685	Prostaglandin A1
	CE6240	9-deoxy-delta12-PGD2

**Table 3:** Composition of the control and experimental high-fat diets used in the experiment on male C57BL/6j mice. All diets were based on the AIN-93G formulation and contained equivalent caloric content. Experimental groups differed in the dominant lipid source: lard (HFD-L), coconut oil (HFD-CO), olive oil (HFD-OO), or cod liver oil (HFD-FO), while the control diet (Ctrl.) included only soybean oil and minimal lard. Macronutrient sources, micronutrient mixes, and fiber content were held constant across all diets.

Feed Composition:	Ctrl.	HFD-L	HFD-CO	HFD-OO	HFD-FO
Corn starch (%)	53.6	18.5	18.5	18.5	18.5
Casein (>85% protein) (%)	21.2	27.0	27.0	27.0	27.0
Maltodextrin (%)	13.2	16.9	16.9	16.9	16.9
Saccharose (%)	7.3	9.3	9.3	9.3	9.3
Soybean oil (%)	2.6	3.4	3.4	3.4	3.4
Lard (%)	2.1	24.9	8.0	8.0	8.0
Coconut oil (%)	0.0	0.0	16.9	0.0	0.0
Olive oil (%)	0.0	0.0	0.0	16.9	0.0
Cod liver oil (%)	0.0	0.0	0.0	0.0	16.9
Fiber (α-cellulose) (g)	50	50	50	50	50
AIN-93G-Mineral Mix (g)	35	35	35	35	35
AIN-93G-Vitamin Mix (g)	10	10	10	10	10
L-cystine (g)	3	3	3	3	3
Choline Bitartrate (g)	2.50	2.50	2.50	2.50	2.50
Tert-butylohydrochinon (TBHQ) (mg)	140	140	140	140	140

**Table 4:** Fatty acid (FA) profiles of the control (Ctrl.) and experimental high-fat diets (HFD) used during the second stage of the in vivo mouse study. Diet abbreviations: HFD-L (lard-based), HFD-CO (coconut-oil-based), HFD-OO (olive-oil-based), and HFD-FO (fish-oil-based). Fatty acid composition expressed as the percentage of each fatty acid relative to the total identified fatty acids. Meaning of abbreviation n.d.—not detected (below the detection limit).

FA Content (% of Total Detected Fatty Acids)						
FA Omega Nomenclature	Common Name	Ctrl.	HFD-L	HFD-CO	HFD-OO	HFD-FO
C6:0	Caproic acid	n.d.	n.d.	0.17	n.d.	n.d.
C8:0	Caprylic acid	n.d.	n.d.	3.47	n.d.	n.d.
C10:0	Capric acid	0.08	n.d.	3.24	0.07	n.d.
C12:0	Lauric acid	0.10	0.06	27.52	0.41	n.d.
C14:0	Myristic acid	1.26	0.16	12.31	0.61	2.97
C14:1	Myristoleic acid	n.d.	n.d.	n.d.	n.d.	0.15
C15:0	Pentadecanoic acid	0.05	n.d.	n.d.	n.d.	0.20
C16:0	Palmitic acid	22.93	38.78	15.03	17.19	15.55
C16:1n10	Sapienic acid	n.d.	n.d.	n.d.	n.d.	0.23
C16:1n9	Elaidic acid	0.32	0.04	0.10	0.15	0.44
C16:1n7	Palmitoleic acid	2.09	0.18	0.65	1.16	5.36
C17:0	Margaric acid	0.29	0.05	0.12	0.17	0.45
C17:1	Margaroleic acid	0.24	0.04	0.08	0.14	0.54
C18:0	Stearic acid	12.66	12.60	8.32	8.00	7.37
C18:1T	Vaccenic acid	0.11	n.d.	n.d.	n.d.	n.d.
C18:1n9	Oleic acid	37.10	38.17	16.58	55.04	24.76
C18:1n3	(15E)-octadecenoic acid	2.78	0.35	0.92	2.18	3.63
C18:2n7	Rumenic acid	n.d.	n.d.	n.d.	n.d.	0.15
C18:2n6	Linoleic acic	16.55	8.52	9.95	12.51	10.25
C18:3n3 (ALA)	α-Linolenic acid	1.66	0.77	1.06	1.36	1.50
C18:3n6 (GLA)	γ-Linolenic acid	n.d.	n.d.	n.d.	n.d.	0.91
C20:0	Arachidic acid	0.19	0.07	0.13	0.37	0.20
C20:1n7	Paullinic acid	0.85	0.10	0.25	0.37	8.77
C20:2n6	Cis-11,14-Eicosadienoic acid	0.46	0.05	0.11	0.10	0.26
C20:3n9	Mead acid	n.d.	0.05	n.d.	0.10	n.d.
C20:4n6	Arachidonic acid	0.30	n.d.	n.d.	0.33	5.87
C20:5n3 (EPA)	Eicosapentaenoic acid	n.d.	n.d.	n.d.	n.d.	3.75
C21:0	Heneicosanoic acid	n.d.	n.d.	n.d.	n.d.	0.31
C22:3	Docosatrienoic acid	n.d.	n.d.	n.d.	n.d.	0.30
C22:5n3	Docosapentaenoic acid	n.d.	n.d.	n.d.	n.d.	0.71
C22:6n3 (DHA)	Docosahexaenoic acid	n.d.	n.d.	n.d.	n.d.	5.18
C24:1n9	Nervonic acid	n.d.	n.d.	n.d.	n.d.	0.20

## Data Availability

The data presented in this study are available in the article. Further inquiries can be directed to the corresponding author.

## Abbreviations

The following abbreviations are used in this manuscript:

HFD	High-fat diet
SFAs	Saturated fatty acids
MUFAs	Monounsaturated fatty acids
PUFAs	Polyunsaturated fatty acids
IL-6	Interleukin 6
MCP-1	Monocyte chemoattractant protein-1
EPA	Eicosapentaenoic acid
DHA	Docosahexaenoic acid
ROS	Reactive oxygen species

## Appendix A

### Feed Conversion Ratio

**Table A1:** Feed conversion ratio (FCR) during the second part (12 weeks) of dietary intervention in control and high-fat diet (HFD) groups. Diet abbreviations: HFD-L (lard-based), HFD-CO (coconut-oil-based), HFD-OO (olive-oil-based), and HFD-FO (fish-oil-based). Results are expressed as grams of feed consumed per gram of body weight gain (±standard deviation). One-way analysis of variance (ANOVA) with Tukey’s multiple comparison post-test was used to determine the significance of effects between the control and HFD groups. FCR values did not differ significantly among the HFD groups. The significance of effect was noted only in the comparisons: Control vs. HFD-L, Control vs. HFD-CO, Control vs. HFD-OO. Thus, the third column presents the adjusted P values between the control group and HFD-L, HFD-CO, HFD-OO groups.

Experimental Group	FCR	Adjusted *p* Value (Control vs. HFD Groups)
Control	108.8 ± 29.0	-
HFD-L	68.7 ± 20.4 *	0.045
HFD-CO	50.1 ± 25.9 *	0.004
HFD-OO	64.8 ± 31.8 *	0.031
HFD-FO	84.5 ± 35.2	0.478

* represents statistically significant differences (*p* ≤ 0.05) from control group.
